# Supplementary material for: Perfusion Parameters in Near-Infrared Fluorescence Imaging with Indocyanine Green: A Systematic Review of the Literature
Source: Life (Basel). 2021 May 11;11(5):433. doi: 10.3390/life11050433 (PMC8151115; doi:10.3390/life11050433)

Table S1. Quality assessment and risk of bias according to the revised Quality Assessment of Diagnostic Accuracy Studies

	Risk of Bias				Applicability Concerns		
Study	Patient Selection	Index test	Reference Standard	Flow and Timing	Patient Selection	Index test	Reference Standard
Abdelwahab, 2019(6)	-	+	?	-	-	-	-
Abdelwahab, 2019(7)	+	+	?	-	-	-	-
Aiba, 2021(8)	-	+	?	-	-	-	-
Amagai, 2020(9)	-	+	-	-	-	-	-
Betz, 2009(10)	-	+	-	-	-	-	-
Betz, 2013(11)	-	+	-	-	-	-	-
Braun, 2013(12)	-	+	-	-	-	-	-
Colvard, 2016(13)	-	+	-	-	-	-	-
D'urso, 2020(14)	-	+	-	-	-	-	-
Fichter, 2019(15)	-	+	?	-	-	-	-
Gerken, 2020(16)	-	+	-	-	-	-	-
Girard, 2019(17)	-	+	-	-	-	-	-
Goertz, 2019(18)	-	+	?	-	-	-	-
Gorai, 2017(19)	-	+	-	-	-	-	-
Hajhosseini, 2021(20)	-	+	?	+	-	-	-
Han, 2018(21)	-	+	-	-	-	-	-
Hayami, 2019(22)	-	+	-	-	-	-	-
Hitier, 2016(23)	-	+	-	-	-	-	-
Holling, 2013(24)	-	+	-	-	-	-	-
Igari, 2013(25)	-	+	-	-	-	-	-
Igari, 2014(26)	+	+	-	-	+	-	-
Ishige, 2019(27)	-	+	?	-	-	?	-
Kamiya, 2015(28)	-	+	-	-	-	-	-
Kamp, 2017(29)	-	+	-	-	-	-	-

Kamp, 2012(30)	-	?	-	-	-	?	-
Kang, 2011(31)	+	+	-	-	?	-	-
Kang 2010(32)	+	+	-	-	?	-	-
Kobayashi, 2014(33)	-	+	-	-	-	-	-
Lang, 2017(34)	-	+	-	-	-	-	-
Maxwell, 2016(35)	+	+	-	-	?	-	-
Mironov, 2019(36)	-	-	-	-	-	-	-
Miyazaki, 2017(37)	-	+	-	-	-	-	-
Mothes, 2009(38)	-	+	-	-	-	-	-
Nakamura, 2017(39)	-	+	-	-	-	-	-
Nishizawa, 2017(40)	-	+	-	-	-	-	-
Patel, 2018(41)	-	+	-	-	-	-	-
Prinz, 2014(42)	-	+	?	-	-	-	-
Regus, 2019(43)	-	+	-	-	-	-	-
Rennert, 2018(44)	-	+	-	-	-	-	-
Rennert, 2019(45)	-	+	-	-	-	-	-
Rother, 2019(46)	-	+	-	-	-	-	-
Rother, 2018(47)	-	+	-	-	-	-	-
Rother, 2017(48)	-	+	-	-	-	-	-
Rother, 2020(49)	?	+	-	-	-	-	-
Schneider, 2011(50)	?	+	-	-	?	-	-
Seinturier, 2020(51)	-	+	-	?	-	-	-
Settembre, 2017(52)	-	+	-	-	-	-	-
Shi, 2015(53)	-	+	-	-	-	-	-
Son, 2019(54)	-	+	-	-	-	-	-
Tanaka, 2015(55)	-	+	-	-	-	-	-

Terasaki, 2013(56)	-	+	-	-	-	-	-
Uchino, 2014(57)	-	+	-	-	-	-	-
Uchino, 2013(58)	-	+	-	-	-	-	-
Venermo, 2016(59)	-	+	-	-	-	-	-
Wada, 2017(60)	-	-	-	-	-	-	-
Woitzik, 2006(61)	?	+	?	-	?	?	-
Yang, 2018(62)	-	+	-	-	-	-	-
Ye, 2013(63)	-	+	-	-	-	-	-
Zhang, 2020(64)	-	+	-	-	-	-	-
Zimmermann, 2012(65)	-	+	-	-	-	-	-

-, Low risk of bias; + high risk of bias; ? risk of bias unclear

Figure S1. Proportion of studies with low, high, and unclear risk of bias according to the revised Quality Assessment of Diagnostic Accuracy Studies.

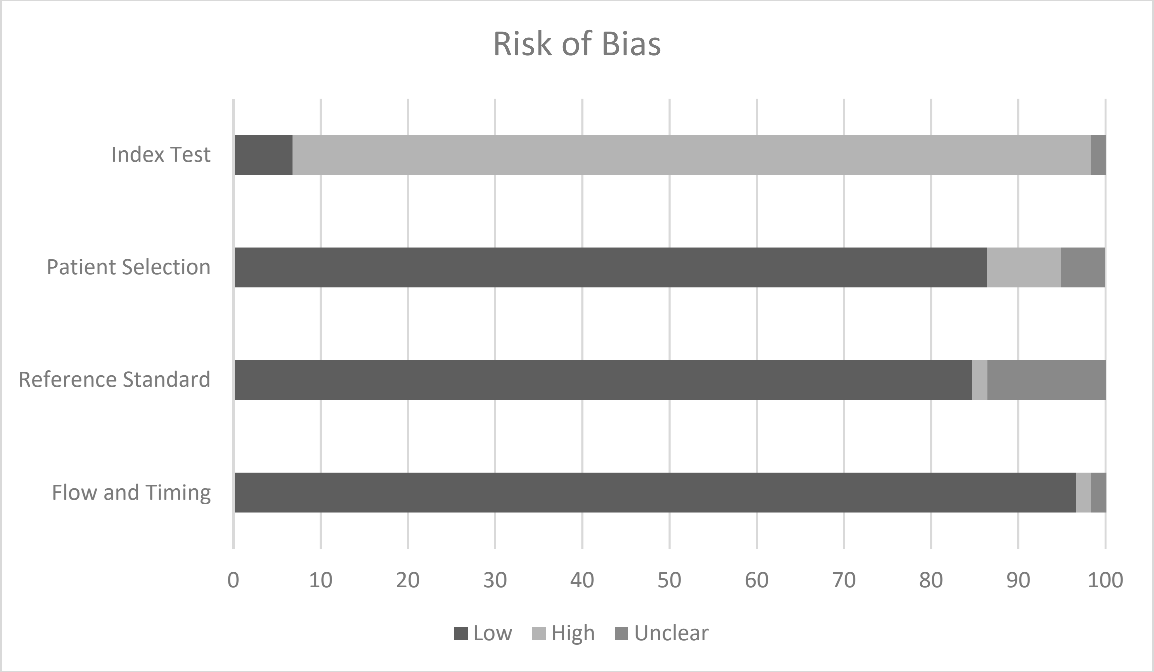

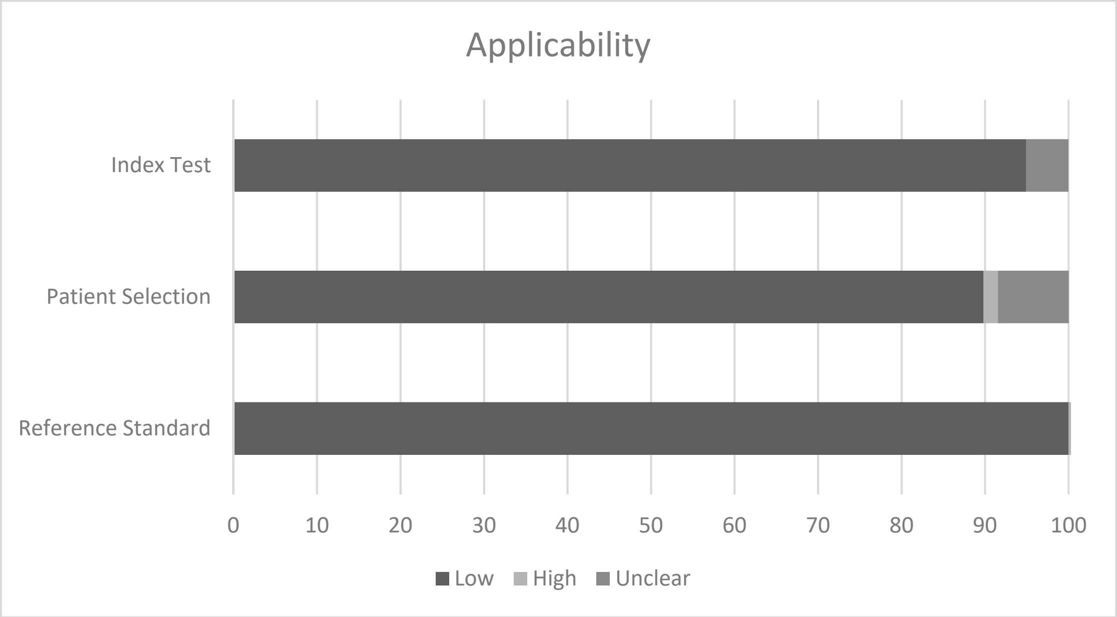

Supplement: Supplementary file 1 [file life-11-00433-s001.zip › life-1207004-supplementary.pdf]
